# Genome-Wide Linkage Scan of a Pedigree with Familial Hypercholesterolemia Suggests Susceptibility Loci on Chromosomes 3q25-26 and 21q22

**DOI:** 10.1371/journal.pone.0024838

**Published:** 2011-10-14

**Authors:** Xu Wang, Xin Li, Yong-Biao Zhang, Feng Zhang, Liyuan Sun, Jie Lin, Duen-Mei Wang, Lu-Ya Wang

**Affiliations:** 1 The Key Laboratory of Remodeling-related Cardiovascular Diseases, Capital Medical University, Ministry of Education, and Beijing Institute of Heart Lung and Blood Vessel Diseases, Beijing Anzhen Hospital of the Capital University of Medical Sciences, Beijing, People's Republic of China; 2 CAS Key Laboratory of Genome Sciences and Information, Beijing Institute of Genomics, Chinese Academy of Sciences, Beijing, People's Republic of China; University of Texas MD Anderson Cancer Center, United States of America

## Abstract

**Background:**

Familial hypercholesterolemia (FH) is a heritable disorder that can increase the risk of premature coronary heart disease. Studies suggest there are substantial genetic heterogeneities for different populations. Here we tried to identify novel susceptibility loci for FH in a Chinese pedigree.

**Methodology/Principal Findings:**

We performed a SNP-based genome-wide linkage scan with the Chinese FH pedigree. Two suggestive linkage loci not previously reported were identified on chromosomes 3q25.1-26.1 (NPL = 9.01, nominal *P*<0.00001, and simulated occurrence per genome scan = 1.08) and 21q22.3 (NPL = 8.95, nominal *P*<0.00001, and simulated occurrence per genome scan = 1.26). In the interaction analysis with a trimmed version of the pedigree, we obtained a significantly increased joint LOD score (2.70) compared with that obtained when assuming the two loci uncorrelated, suggesting that more than one locus was involved in this pedigree. Exon screening of two candidate genes *ABCG1* and *LSS* from one of the suggestive region 21q22 didn't report any causative mutations.

**Conclusions/Significances:**

These results confirm complex etiologies and suggest new genetic casual factors for the FH disorder. Further study of the two candidate regions is advocated.

## Introduction

Familial hypercholesterolemia (FH) is a heritable disorder characterized by high concentration of total cholesterol and low density lipoprotein cholesterol in serums. Elevated levels of cholesterol can give rise to xanthomas, a deposit of cholesterol in peripheral tissues, accelerating atherosclerosis, and therefore increase the risk of premature coronary heart disease (CHD) [1]. The FH phenotype is affected by both environmental and genetic factors [2].

The majority of FH cases are transmitted in an autosomal-dominant manner, known as autosomal dominant hypercholesterolemia (ADH or FH, MIM ID #143890). Three genes identified in autosomal dominant FH disorder are the low density lipoprotein receptor gene (*LDLR*) on chromosome 19 [3], the apolipoprotein B gene (*APOB*) on chromosome 2 [4], and the proprotein convertase subtilisin/kexin type 9 gene (*PCSK9*) on chromosome 1 [5]. Other FH loci currently listed in OMIM include 1q21-q23, 9q22-q31, 8p21-p12, 7p15, 5p13-p12, 3p21.2-p14.1, and 19p13.2 (MIM ID #143890). Autosomal recessive inheritance, referred to as autosomal recessive hypercholesterolemia (ARH, MIM ID #603813), was also observed in some pedigrees. Thus far only one ARH locus, 1p36-35 [6], [7], is listed in OMIM, although two other loci for ARH, located on 13q22-32 [7] and 15q25-26 [8], have also been reported.

Genetic heterogeneity for FH among different populations provides the possibility of uncovering other uncharacterized genes that may be involved in the pathogenesis of the disease. For example, in a Mexican population, no *PCSK9* mutations were found in one large FH family that showed positive linkage to the 1p34.1-32 locus; this indicates genes other than *PCSK9* in the locus may be involved [9]. In addition, a Portuguese FH study found only 48% of its total received cases with clinical diagnosis of FH had genetic defects on *LDLR*, *APOB* or *PCSK9*, leaving the other 52% of FH cases with possible undiscovered gene mutations [10].

To identify novel FH loci in Chinese individuals, we performed a genome-wide linkage analysis of a Chinese pedigree without the known FH mutations in *LDLR*, *APOB*, and *PCSK9*, using an Illumina Human Linkage-12 panel, which contains 6,090 SNP markers with an average spacing of 0.58 cM. A two-locus linkage (assuming two disease loci) analysis was performed to identify linkage signals and potential locus interaction.

## Results

### Plasma lipid concentrations and clinical characteristics

**Figure 1:**
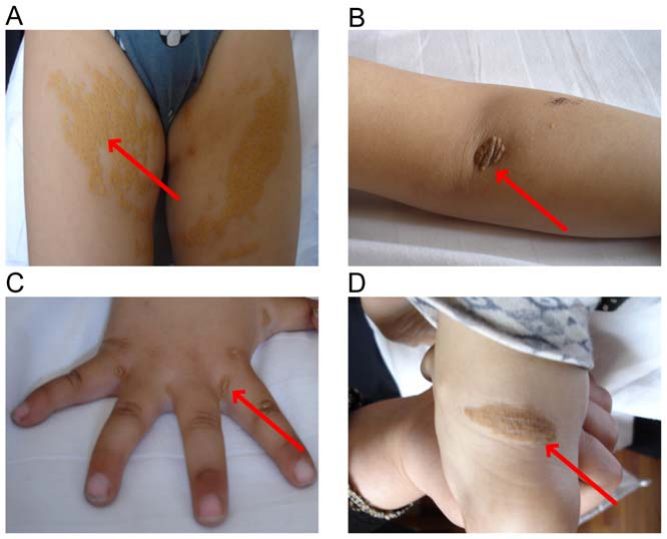
Clinical manifestations of the proband that had a typical FH phenotype (top panel). (A) Cutaneous xanthomas of buttocks, (B) Cutaneous xanthomas on elbow region, (C) Tendinous xanthomas and small cutaneous xanthomas of the hands, (D) Achilles tendon xanthomas.

Lipid levels and clinical features including xanthoma and coronary heart disease (CHD) for the pedigree members are shown in Table 1. Without lipid-lowering drug therapy, the plasma total cholesterol levels of these patients ranged from 226.9 to 817.2 mg/dl and the levels of LDL cholesterol ranged from 122.6 to 761.1 mg/dl. Clinical manifestations of the proband are shown in Figure 1. Patient 209 died of coronary heart disease at 45 years old and patients 2 and 34 developed coronary heart disease. The remaining members of the pedigree showed no abnormal signs of related diseases, such as coronary heart disease, diabetes mellitus, thyroid disorder, or hepatorenal disease. All pedigree members had undergone a bland diet without smoking or drinking alcohol.

**Table 1:** Plasma lipid concentrations and clinical characteristics.

ID	Sex	Age	TC	TG	LDL-C	HDL-C	ApoAI	ApoB	Xanthomas	CHD	FH diagnosis
		(years)	(mg/dl)	(mg/dl)	(mg/dl)	(mg/dl)	(g/L)	(g/L)			
1	Female	46	141.6	224.8	45.5	51.1	1.17	0.69	no	no	unaffected
2	Female	54	226.9	132.4	141	59.4	1.31	0.87	no	yes	affected
3	Male	56	146.7	132.4	62.6	57.6	1.28	0.71	no	no	unaffected
7	Female	28	290.6	270.3	166.5	70	1.47	0.68	no	no	affected
21	Male	33	102	129.4	31.4	44.7	1.24	0.55	no	no	unaffected
22	Male	32	148	134.8	60	61.1	1.55	0.62	no	no	unaffected
24	Female	57	317.9	195.6	220	58.8	1.04	0.79	no	no	affected
27	Male	32	146.1	100.9	62.1	63.8	1.35	0.58	no	no	unaffected
28	Female	26	104.2	65.5	29.1	62	1.25	0.46	no	no	unaffected
34	Male	50	260.2	262.6	152.1	55.6	1.27	1.04	no	yes	affected
35	Male	25	228.2	92.4	133.3	76.4	1.09	0.51	no	no	affected
36	Male	33	241.8	212.7	122.6	36.7	1.19	1.21	no	no	affected
37	Female	30	356.6	83.2	285.9	54.1	1.13	1.24	no	no	affected
38	Male	5	817.2	113.2	761.1	33.5	0.77	0.99	yes	no	affected
40	Female	48	290.6	148.6	176.2	84.7	1.49	0.85	no	no	affected
41	Male	22	241.2	129.4	58.2	157.1	1.29	0.94	no	no	affected
45	Female	30	285.6	123.9	186.4	74.4	1.18	0.89	no	no	affected

### Genome-wide linkage analysis

**Figure 2:**
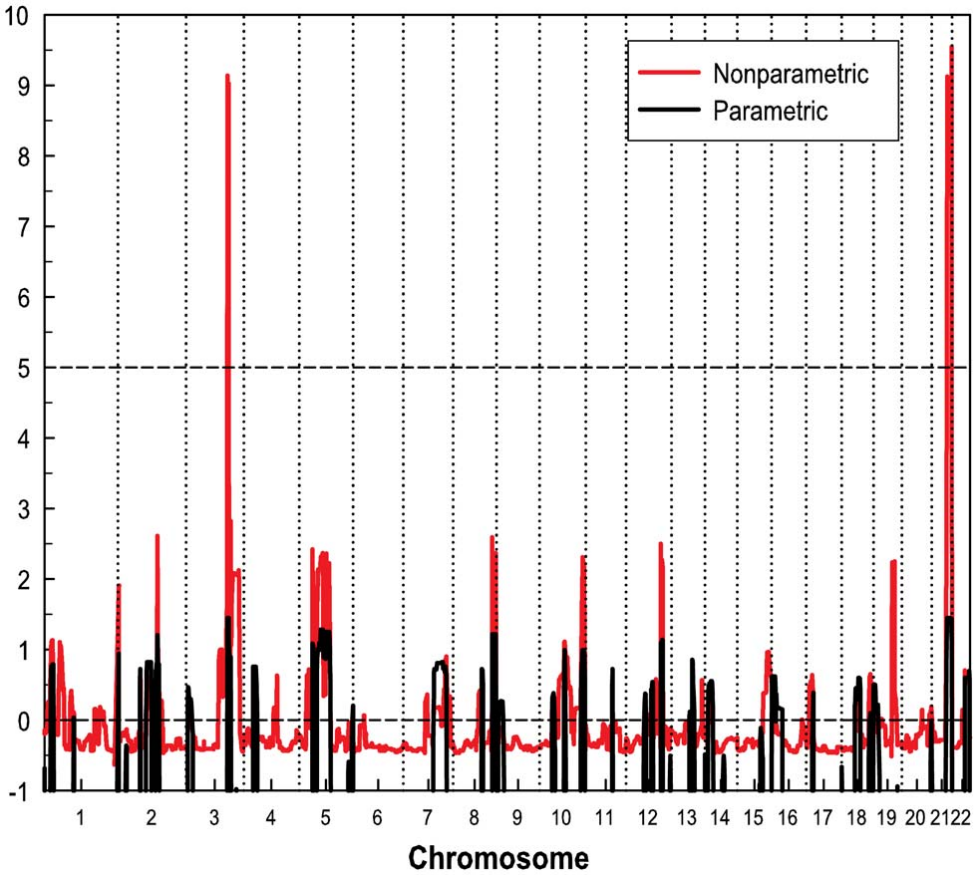
Genome-wide parametric and nonparametric linkage results of the FH pedigree. In the parametric linkage analysis, an autosomal dominant model with a risk allele penetrance of 0.2 and a phenocopy rate of 0.0001 was assumed.

The nonparametric genome-wide scan revealed two prominent signals with nonparametric linkage (NPL) scores of 9.01 (nominal *P*<0.00001, and simulated occurrence per genome scan = 1.08) and 8.95 (nominal *P*<0.00001, and simulated occurrence per genome scan = 1.26) on chromosomes 3q25-26 and 21q22, respectively (Figure 2), while no other genomic regions showed NPL scores greater than 5. The significance levels of these two NPL signals were of proximity to the standard of suggestive linkage proposed by Lander & Kruglyak [11], where suggestive linkage is statistical evidence that would be expected to occur one time at random in a genome scan. The two highest LOD scores (both were 1.45) of parametric genome-wide linkage analysis coincided with the two NPL peak regions on chromosomes 3q25-26 and 21q22.

No linkage signals were found in those previously reported regions for either autosomal dominant or recessive FH. The two regions with prominent linkage signal, chromosomes 3q25-26 and 21q22, were used for further interaction analysis.

### Haplotype analysis

**Figure 3:**
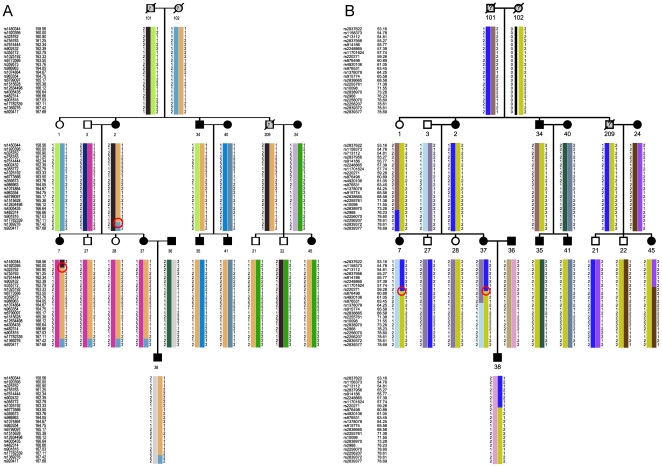
The most likely haplotypes of the FH pedigree on chromsomes 3q25-26 (A) and 21q22 (B). The black symbols indicated the affected individuals. The red circles indicated critical recombinations. Marker names and their positions (cM) are listed on the left side.

To define the minimal co-segregating intervals on chromosomes 3q25-26 and 21q22 in our affected individuals, haplotypes were constructed in the relevant genomic regions. The centromeric boundary of the interval on 3q25-26 was defined by a recombination event between SNP markers rs1920395 and rs325762, which was observed in individual 7. The telomeric boundary of this interval corresponded to a historic recombination event between rs17782339 and rs1369276 in individual 2. These recombination events defined the linkage signal on 3q25-26 to a 7.42 cM interval (160.00–167.42 cM; 152.45–165.64 Mb) between rs1920395 and rs1369276 (Figure 3A). On chromosome 21q22, the centromeric boundary was defined by recombination events between rs220271 and rs876498 in individuals 7 and 37. Thus, a 20-cM interval (59.28 cM-telomere; 42.36–46.90 Mb) on chromosome 21 from rs220271 to the q telomere was defined (Figure 3B). All the affected members shared both of the disease linked haplotypes on 3q25-26 and 21q22. With these data from our FH pedigree, we refined the linkage signals of the two susceptibility loci to 3q25.1-26.1 and 21q22.3, respectively.

Notably, the disease linked haplotype on either locus was also shared among some unaffected individuals. On chromosome 3q25-26, those individuals were 27, 28, and 21 (Figure 3A). On chromosome 21q22, they were 1, 28, and 22 (Figure 3B). This phenomenon suggests a possible incomplete penetrance of either susceptibility locus.

### Two-locus linkage analysis

The two-locus linkage analyses on 3q25-26 and 21q22 further improved the linkage scores in both parametric and nonparametric analyses over those from single-locus analyses. The two-locus linkage analysis using a trimmed version of the pedigree (without individuals 35, 40, and 41) under a multiplicative model yielded a maximum joint LOD score of 2.70. This was a significant increase over the single-locus LOD scores of 1.10 and 1.14 on chromosomes 3q25-26 and 21q22, respectively, with the same trimmed pedigree structure. It was also observed that, the parametric two-locus linkage analyses using a heterogeneous and an additive model yielded LOD scores of 1.17 and 1.19, respectively, which are no better than the single-locus LOD scores with the same trimmed pedigree structure. Therefore, the interaction between these two regions was better modeled by the multiplicative model. This interaction was further supported by a joint NPL score (6.47; *P* = 0.000565) that was higher than those from single-locus analysis on chromosomes 3q25-26 and 21q22 (4.09 and 4.47, respectively).

### Candidate gene sequencing

We sequenced all the exons and intron-exon boundaries of two most likely candidate genes *ABCG1* and *LSS* on 21q22.3, using DNA samples from 8 selected individuals of the pedigree. Five SNPs were detected in the exons of *LSS*, including 2 synonymous SNPs, rs11558754 and rs2254522, and 3 nonsynonymous SNPs, rs2254524, rs34115287, and rs2839158. No SNPs were observed in the exons of *ABCG1*, however 6 SNPs were detected in the introns of *ABCG1*. No *de novo* mutations or indels were found in the sequenced regions of these genes. None of the observed SNPs was related to the FH phenotype or was reported from HapMap to be associated with diseases.

## Discussion

Based on a genome-wide linkage scan of a Chinese pedigree using SNP markers, we identified two suggestive linkage loci for FH. One locus was mapped to 3q25.1-26.1 in a 7.42-cM interval (13.19 Mb) between rs1920395 and rs1369276, and the other was mapped to 21q22.3 in a 20-cM interval (4.54 Mb) from rs220271 to the q telomere. Both regions are distinct from the previously reported candidate regions for both autosomal dominant and recessive FH. Although the parametric linkage scores for both loci were not significant, the nonparametric scores were suggestively significant for both loci (nominal *P*<0.00001, with an occurrence of about 1 time per genome scan). The main reason for the insignificance of the parametric linkage score was the difficulty in defining an accurate mode of inheritance. Incomplete penetrances, phenocopies, or polygenic inheritance may also lead to loss of power in single-locus parametric linkage mapping [12]. Non-parametric linkage analysis has been described as a powerful approach that can account for the possibility of alternative modes of inheritance, and has been referred to as the method of choice for pedigree studies of complex traits [13].

The two-locus model can greatly improve the power to detect linkage for traits governed by two or more loci. The interaction between the two uncovered loci on chromosomes 3q25-26 and 21q22 was suggested by both parametric and nonparametric two-locus linkage analyses. Two-locus linkage analysis has been successfully used to detect susceptibility loci for familial hypercholesterolemia [7] and other complex traits, such as familial combined hyperlipidemia [14], high factor VIII (FVIII) levels in blood [15] and bipolar affective disorder [16]. In these studies, the superiority of the two-locus linkage scores compared to the single-locus scores was considered as evidence of genetic interaction. It is regarded that the greater scores of two-locus linkage yields, the stronger interaction between the examined loci. Our two-locus linkage analysis supported the two linkage signals, and suggested a multiplicative interaction between the two loci. The observed haplotype transmission was also in agreement with the assumption of two disease loci, because it reduced the number of unaffected disease-linked haplotype carriers from 3 for single locus (either 3q25-26 or 21q22) to 1 for two loci (Figure 3). Thus, the genome-wide linkage scan indicated a different and complex genetic basis of FH for this pedigree compared with those previously reported.

There are 92 and 113 genes in the candidate regions of chromosomes 3q25.1-26.1 and 21q22.3, respectively. We selected the two most likely candidate genes *ABCG1* and *LSS* for a preliminary mutation screening. Both genes are located on 21q22.3. *ABCG1* is a key gene involved in both cholesterol efflux to HDL and tissue lipid homeostasis [17]. ABCG1 and ABCG4 act in concert with ABCA1 to maximize the removal of excess cholesterol from cells and to generate cholesterol-rich lipoprotein particles [18]. *LSS* encodes an enzyme that catalyzes the first step of cholesterol biosynthesis, the conversion of (S)-2, 3 oxidosqualene to lanosterol. Exon screening of both genes failed to identify any mutation that co-segregated with the disease trait in this pedigree. This result was not unexpected, because more than one gene was implied in the pedigree according to our results. It is more probable that a few common variants, each with mild effect rather than a single causative mutation, may contribute to FH. Nevertheless, detailed screening for molecular variants in the candidate genes should be carried out, perhaps with increased screening size in a case-control design, and including extended screening regions beyond exons. Indeed, recently a novel *ABCG1* −257T>G promoter polymorphism that influences CHD severity in Japanese males has been found [19].

In this study, the proband had a characteristic FH phenotype with extremely high levels of plasma TC (817.2 mg/dl) and LDL-C (761.1 mg/dl) and tendinous xanthomas, while the other affected members had a relatively mild phenotype. Because both parents were affected, one possibility was that the proband may inherit two sets of pathogenic mutations (i.e., the maternal and paternal mutations) in one gene or in different genes. Previous clinical observations revealed that patients with compound heterozygote *LDLR* mutations can exhibit a more severe FH phenotype than their parents who carried only a single mutation [20]. Unfortunately, the proband's paternal family members were unavailable for the linkage study. An intensive study of the family members with next-generation sequencing technology should be helpful in indentifying the molecular basis of the phenotype.

In summary, the SNP-based genome-wide linkage scan with a Chinese FH pedigree revealed two suggestive linkage signals on chromosomes 3q25.1-26.1 and 21q22.3. Both loci are distinct from previously reported FH regions. Interaction analysis assuming two disease loci suggested the involvement of more than one locus in this pedigree. *ABCG1* and *LSS* were identified as plausible candidate genes in one of the candidate regions. Our findings revealed new and complex genetic etiology for the disease. Further research is advocated to identify the susceptibility genes in these newly discovered candidate regions.

## Materials and Methods

### Ethics Statement

The study complies with the Declaration of Helsinki. All participants provided written consent, and the ethics committee of the Beijing Anzhen Hospital of the Capital University of Medical Sciences approved all studies. Separate informed consent was obtained to publish the photographs in Figure 1 from the guardian of the proband.

### Pedigree ascertainment and description

A 5-year-old boy with cutaneous vegetations on bilateral elbows, knees, and buttocks was identified as the proband. A further examination at the Beijing Anzhen Hospital found multiple cutaneous and tendinous xanthomas in the boy (Figure 1). The concentrations of plasma total cholesterol (TC) and LDL-cholesterol (LDL-C) of the proband were 817.2 mg/dl and 761.1 mg/dl, respectively. Doppler ultrasound revealed increased intima-media thickness and multiple atherosclerotic plaques in the common carotid artery, right subclavian artery, abdominal aorta, and femoral artery of the proband, especially in bifurcate region and posterior walls of arteries.

An extended four-generation pedigree of the proband with 17 available members from Henan Province, China was obtained for this study. All individuals had their lipoprotein measurements recorded and general information such as gender, age, history of smoking, dietary habits, personal history, and family history were collected at the same time (Table 1). None of the parents were in a consanguineous marriage. Individuals were evaluated clinically by at least two cardiologists. In total, 11 individuals (5 males and 6 females) were diagnosed as FH-affected. All the affected individuals, except individual 2, meet the criteria suggested by Williams [21]. Individual 2 was diagnosed as “affected,” based on her borderline high TC concentration (226.9 mg/dl), family history, and positive status of CHD.

### Serum lipids measurements

Venous blood samples were drawn from the pedigree members after overnight fasting for 12 to 14 hours. The concentrations of total plasma cholesterol, triglycerides, and high-density lipoprotein cholesterol (HDL-C) were measured by applying standard enzymatic techniques. The LDL-C concentration was calculated by applying the Friedewald formula [22]. Levels of Apo AI and Apo B100 were measured by ELISA.

### Genotyping

Genomic DNA was extracted from venous blood of the 17 available pedigree members using a phenol-chloroform method. Prior to the linkage scan, we excluded the possibility that any known mutations from the three FH genes, *LDLR*, *APOB*, and *PCSK9*, caused the FH phenotype of this pedigree. The *LDLR* gene was examined by sequencing, whereas the *APOB* and *PCSK9* loci were examined by genotyping locus-linked microsatellite markers.

The genome-wide linkage scan was then conducted using the Illumina Human Linkage-12 panel. The panel contains 6,090 single nucleotide polymorphism (SNP) markers that are well spaced. Reactions were performed according to the manufacturer's protocols. Fluorescent signals were scanned using Illumina's BeadStation; genotypes were called with Illumina BeadStudio Software v3.1.8.

### Genome-wide linkage analysis

Mendelian inconsistencies of the genotype data were investigated with PEDCHECK v1.1 [23]. Unlikely double recombinants were identified with the genotype error option of the linkage software MERLIN v1.01. In our linkage analyses, either monomorphic or non-Mendelian transmitted markers were removed before analysis. Of the 6090 SNPs gentoyped, 5170 informative autosomal markers were used for linkage analysis. The average genetic and physical distances between these markers were 0.69 cM and 557 kb, respectively. Both parametric and non-parametric linkage analyses were performed using the linkage software MERLIN v1.01 [24]. Parametric linkage analysis was under an autosomal-dominant model with the following parameters: a risk allele frequency of 0.001, an incomplete penetrance of 0.2 for genotypes with 1 or 2 copies of the risk allele, and a phenocopy rate of 0.0001. The low penetrance 0.2 was chosen for the following two reasons: (i) the model with a penetrance of 0.2 gave higher LOD scores than the other tested models for both the NPL-peak regions of the genome; and (ii) the disease transmission patterns in the pedigree, including the existence of affected marry-ins, unaffected offspring sharing putative disease-locus-linked haplotype, and unknown affection status for deceased individuals, indicated a low penetrance. The critical recombination events of the pedigree members were determined through haplotype construction in MERLIN v1.01.

### Power estimation and simulation

The pedigree used in our study contained 17 genotyped individuals with 11 affected and 6 unaffected members. Power calculation was performed with SLINK [25], assuming an autosomal-dominant model with penetrance of 0.95 and a phenocopy rate of 0.001. The power to detect LOD scores greater than 1 and 2 were 80% and 51%, respectively.

To determine the genome-wide significance level of the linkage signal, we performed simulation studies of 1000 replicates generated with the gene-dropping approach implemented in MERLIN. In each simulation, marker allele frequencies, genetic distances, pedigree structures, and missing data patterns were retained. The number of simulations exhibiting equal or greater linkage scores was used to calculate the frequency of the observed linkage signal occurring by chance.

### Two-locus linkage analysis

To test for an interaction between two putative loci on different chromosomes, GENEHUNTER-TWOLOCUS software was used to perform parametric and nonparametric linkage analysis under two disease loci [26]. Three classes of interaction models (heterogeneity, multiplicative, and additive) were tested according to the method described by Strauch et al. The heterogeneity model assumes that a mutation at either locus can cause the phenotype by itself (“or” condition). The multiplicative model assumes that only mutations at both loci can cause the phenotype (“and” condition). As for the additive model, penetrances of a single locus are simply added and rescaled to form two-locus penetrances. Table 2 shows the two-locus penetrance we used in this analysis. Because the two-locus analysis is computationally-intense, to speed up the interaction test, the pedigree was trimmed by removing the least-informative individuals 35, 40, and 41.

**Table 2:** Penetrance for two-locus linkage analysis.

Model	risk genotype for single locus	risk genotype for both loci
Heterogeneity	0.1997	0.3597
Multiplicative	0.0005	1.0000
Additive	0.1996	0.3995

### Candidate gene sequencing

Five affected and 3 unaffected pedigree members, including the proband, his parents, his maternal uncles and aunts, and his maternal grandparents, were selected for sequencing of the candidate gene. All exons and intron-exon boundaries of the two candidate genes, *ABCG1* (15 exons) and *LSS* (23 exons) were amplified with PCR and sequenced on ABI Prism 3730xl DNA Analyzer.
